# Discovery of a potential open ocean nursery for the endangered shortfin mako shark in a global fishing hotspot

**DOI:** 10.1038/s41598-025-85572-4

**Published:** 2025-01-16

**Authors:** Gonzalo Mucientes, Alexandre Alonso-Fernández, Marisa Vedor, David W. Sims, Nuno Queiroz

**Affiliations:** 1 Centro de Investigação em Biodiversidade e Recursos Genéticos, CIBIO-InBIO, Universidade do Porto, Campus Agrário de Vairão, r/ Padre Armando Quintas, Vairão, 4485-661 Portugal; 2 BIOPOLIS Program in Genomics, Biodiversity and Land Planning, CIBIO, Campus de Vairão, Vairão, 4485-661 Portugal; 3 Instituto de Investigaciones Marinas (IIM), CSIC, Eduardo Cabello 6, Vigo, 36208 Spain; 4 Marine Biological Association, , The Laboratory, Citadel Hill, Plymouth, PL1 2PB UK; 5 Ocean and Earth Science, University of Southampton, National Oceanography Centre Southampton, Waterfront Campus, Southampton, SO14 3ZH UK

**Keywords:** Conservation, Fisheries management, *Isurus oxyrinchus*, Nursery, Size distribution, South Pacific, Developmental biology, Ecology, Zoology

## Abstract

Populations of large pelagic sharks are declining worldwide due to overfishing. Determining the overlap between shark populations and fishing activities is important to inform conservation measures. However, for many threatened sharks the whereabouts of particularly vulnerable life-history stages – such as pregnant females and juveniles – are poorly known. Here, we investigated the spatial distribution of size classes, energy transfer and reproductive states of pregnant females of the endangered shortfin mako, *Isurus oxyrinchus*, using spatially resolved catch data from a Spanish surface longline vessel (1996 − 2009) in the South-east Pacific Ocean. Our results suggest a general eastward gradient of occurrence of pregnant females of thousands of kilometers from western oceanic feeding grounds towards the eastern Pacific, where we observed an aggregation area of small juveniles. Moreover, the potential nursery likely overlapped a longline fishing hotspot, increasing the vulnerability of juveniles from fisheries. Our results suggest that limiting fishing pressure in this area could reduce mortality of early life stages and contribute to the conservation of this endangered shark species.

**Supplementary Information:**

The online version contains supplementary material available at 10.1038/s41598-025-85572-4.

## Introduction

Oceanic sharks are fished intensively worldwide^1,2^ and their relative abundance has declined by ~ 70% since 1970^3^. Moreover, industrialized fishing effort is often high in open ocean areas where subadult (young-of-the-year/juveniles) populations of oceanic sharks aggregate^1,2,4–7^. Importantly, mortality in these age classes is considered the most critical factor in shark population recruitment^8,9^. Population growth of elasmobranchs is limited by their low reproductive potential, low fecundity (large species exhibit long and complex reproductive cycles, frequently lasting more than one year) and slow embryonic and post-embryonic development^10–13^. Oceanic sharks have, however, developed some adaptations to increase reproductive success, enhance offspring survival and reduce the risk of predation of neonates^12,14^. Reproductive strategies include, among others, aplacental viviparity with oophagy, where pregnant females gradually transfer the energy stored in their enlarged livers, to embryos via unfertilized egg during the different developing stages^13,15,16^. Hence, maternal features (e.g., size and age) and offspring size are often positively correlated^17^. Moreover, the energetic costs of reproduction and the ability to restore energy after reproduction are linked to both survival and thus, the capability to continue reproducing^18–20^. Therefore, the study of how pelagic sharks allocate energy resources is fundamental to understand reproductive success^21–23^. On the other hand, several shark species have developed a range of strategies, including the use of nurseries^4,24–27^, to enhance offspring survival rates^28^. In general, shark nurseries have been characterized by: (*i*) higher probabilities of encountering juveniles than in adjacent areas, (*ii*) above average site fidelity; and (*iii*) repeated use between years^26,29^, in addition to (*iv*) abundant prey resources and (*v*) reduced predation risk^30–32^ e.g., where smaller sharks are isolated from adults^6,30,33–38^. The current understanding of elasmobranch nurseries is, however, biased towards tropical coastal shark species with few studies on oceanic species^6,26,29,39,40^. Since juvenile mortality is considered one of the most critical factor impacting the sustainability of shark populations^26,39,41^, high fishing effort in nurseries will contribute to faster population declines^3,42–46^. Therefore, the availability of information to support well-protected and managed nursery (and other core) areas for the species productivity, is likely to benefit shark populations^26,39^.

The degree of overlap of the different species with fishing hotspots, and in particular, the overlap between fishing and the different components of the populations (e.g. mature vs. immature or males vs. females) has only recently received attention^1,2,47^. It was recently demonstrated, at an ocean-basin scale, that the catch-per-unit-effort of pelagic sharks by surface longliners was significantly greater in areas in which the spatial overlap intensity between pelagic shark space use and longline fishing effort was also higher^2,48–50^. While management and conservation measures have been implemented to attempt to recover oceanic shark populations, e.g. retention bans^51–55^, it is evident that bycatch rates currently remain too high for many species to rebuild populations^48,53,56^. Much of that bycatch is due to the extensive spatio-temporal overlap of sharks and fishing effort that is largely aimed at target species which occupy similar habitats to sharks^1,2^. Improving the basic knowledge of shark ecology, spatial patterns, reproduction biology and their life cycle is therefore essential for conservation and management purposes, particularly determining the locations of core areas for the species reproduction (e.g., parturition/nursery areas, juvenile hotspots) in relation to anthropogenic threats^26,39^.

The shortfin mako (*Isurus oxyrinchus*) is an oceanic pelagic species with a circumglobal distribution in tropical and temperate seas around the world^57^ with extensive movements^2,58–65^, including trans-oceanic migrations^62^. The birth size is around 65–80 cm TL (total length), males reach sexual maturity at 200 cm TL, and females at 280 cm TL^10^. The mode of reproduction is aplacental viviparity (matrotrophy) with oophagy, where the embryos in the uterus are fed by unfertilized eggs produced by the mother; however, in general, the reproductive ecology of lamnoids is still poorly understood^66–68^. Particularly, the net energetic transfer during the embryonic development has not yet been quantified for shortfin mako sharks. Moreover, where females give birth and where young-of-the-year and juveniles may aggregate remains poorly described^69^. Few shortfin mako nursery areas have been identified throughout its range^35,59,70,71^; in the North Pacific Ocean, two nurseries have been identified in coastal waters: the Southern California Bight, along the U.S. and Mexican coasts^35,70,72^, and Bahia Sebastian Vizcaino (Baja California, Mexico)^35,71^. In addition, coastal waters of Japan were described as hotspots for immature individuals^73^.

Shortfin mako has experienced population declines between 30 and 50% globally, being one of the most frequently caught pelagic shark in open-ocean longline fisheries^2,3,74^ and the subject of stock assessments^75–78^. There are two stocks in the Pacific Ocean, north and south, separated by the Equator; the South Pacific includes, in turn, a southwest and south-eastern stock^79^. In the North Pacific Ocean stock assessments (in 2018) concluded that the population was not subject to overfishing and was not overfished^80^, while other authors suggested that it might have been overexploited based on current estimated fishing mortality levels^77^. In the South Pacific, shortfin mako shows a clear spatial sexual segregation pattern^47^, and also spatial segregation between pregnant females and different size/age class^69,73,81^. In turn, this could result in biased vulnerability to capture from fisheries^74,82^. Given the significant uncertainty about the locations of shortfin mako nurseries in the open-ocean and international open waters, the quality of stock assessments may be undermined, thus posing difficulties in identifying effective management measures underpinned by scientific evidence^75,76,78,79^.

To address the knowledge gaps on shortfin mako nurseries we used spatially resolved catch data from a Spanish surface longline vessel (1996 − 2009) in the South-east Pacific Ocean to investigate (*i*) the spatial distribution of size classes and (*ii*) evaluate the risk critical life stages from fisheries exposure. Moreover, anatomical measurements were used to determine (*iii*) energy transfer and reproductive states of pregnant females. This enabled the evaluation of the risk critical life stages are subjected from fisheries and provided the first evidence of an open-ocean potential nursery area for endangered shortfin mako sharks in the south-east Pacific at risk from fisheries, which will inform management plans.

## Materials and methods

### Logbook catch data

**Fig. 1:**
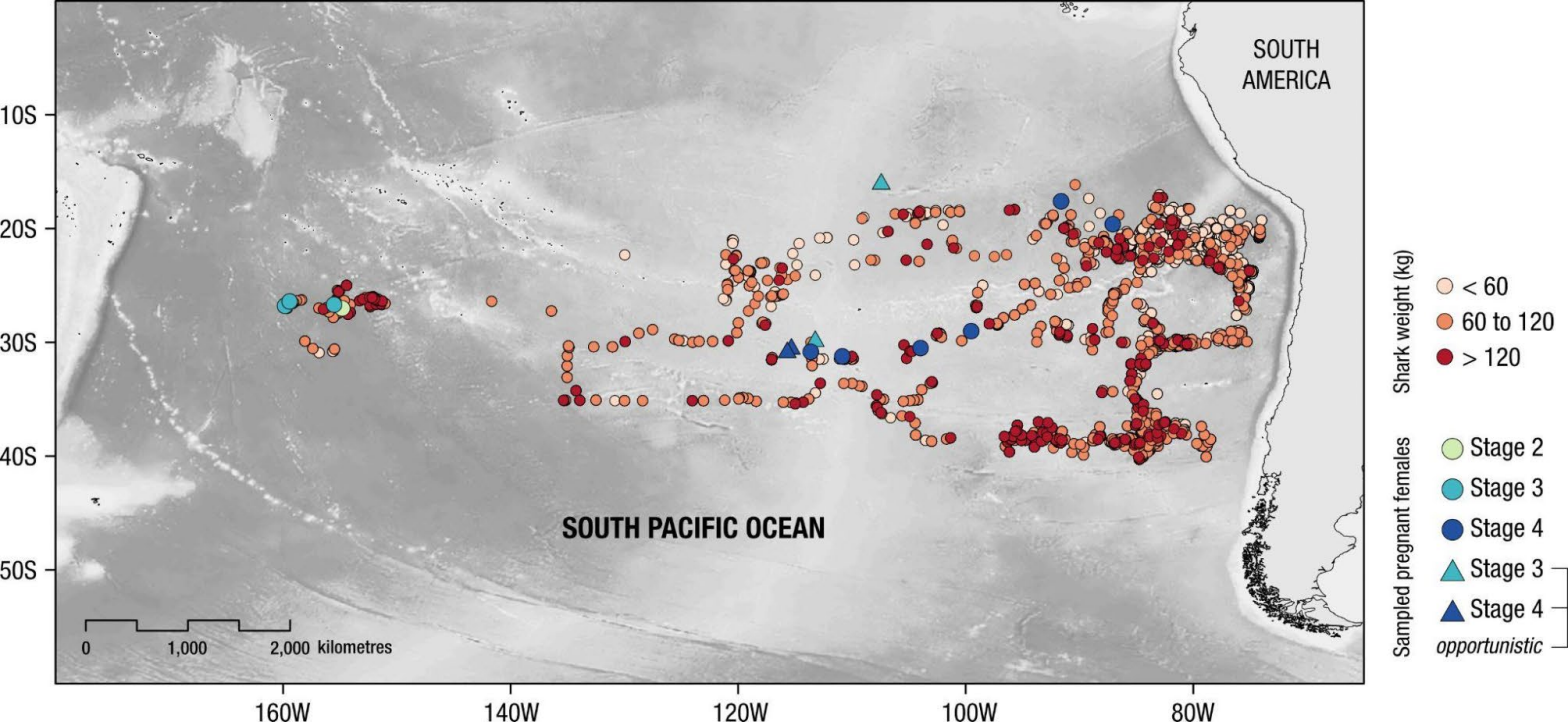
Geographic positions of shortfin mako *Isurus oxyrinchus*, caught in a commercial longline fishing vessel between the years 1996–2009 coloured by their respective weight class (red gradient dots), and locations of captured pregnant females coloured by embryo development stage (blue gradient symbols), in the South Pacific Ocean. *Opportunistic* denotes further observations of pregnant females that were sampled by the crew of the longliner in 2011, 2012 and 2020 (triangles). Map created using ESRI ArcGis Pro software (version Pro 3.3).

Between 1996 and 2009 a total of 24 fishing trips (~ 3 months duration each) and 1,789 longline sets (one set is a single deployment and retrieval of the longline) were conducted in the South Pacific Ocean by a single Spanish longline vessel (Fig. 1; Table 1). The longline deployed by this vessel was ~ 100 km in length with ~ 1,000 baited hooks set at a depth between 40 and 80 m^83^. For each individual shark caught during this fishing period, we obtained information on date, location (latitude and longitude) and weight. To estimate maturity, a combined sexual maturation size of ~ 60 kg (175 cm FL;^84^ was established, since the sex of the sampled individuals was unknown; below this threshold both sexes are unequivocally considered juveniles. Sharks between 60 and 120 kg were considered small adults, and above 120 kg, large adults.

**Table 1:** Summary table of logbook data of shortfin mako, *Isurus oxyrinchus*, catches between 1996 and 2009. SD = standard deviation.

Year	Weight (kg)	Sharks (*n*)	Average individual mass (kg, mean ± SD )	Hooks (*n*)	CPUE (kg/1000 hooks)	CPUE (*n*/1000 hooks)
1996	30,806	710	43.39 ± 22.56	82,800	372.05	8.57
1998	103,726	3320	31.24 ± 19.00	304,300	340.77	10.91
1999	70,826	1853	38.22 ± 23.45	511,500	138.47	3.62
2000	58,109	1343	43.27 ± 28.33	524,700	110.75	2.56
2001	39,842	1782	22.36 ± 16.79	172,865	230.48	10.31
2003	58,204	1549	37.58 ± 30.10	430,100	135.33	3.60
2004	64,235	1374	46.75 ± 34.02	478,452	134.26	2.87
2005	56,482	1160	48.69 ± 31.41	443,440	127.37	2.62
2006	61,149	925	66.11 ± 35.63	403,900	151.40	2.29
2007	17,370	339	51.24 ± 29.73	209,400	82.95	1.62
2008	21,604	755	28.61 ± 17,56	305,850	70.64	2.47
2009	30,776	966	31.86 ± 25.17	207,600	148.25	4.65
Total	613,129	16,076	38.14 ± 27.76	4,074,907	150.46	3.95

### Longline fleet fishing effort data

We used location data from both the Vessel Monitoring System (VMS) and Automatic Identification System (AIS). VMS data for the Spanish fleet operating in the operating in the south-east Pacific region was obtained for the years 2010 and 2011 and the number of fishing days calculated for each 1 × 1° grid cell following the methods in Queiroz et al., (2016). The mean fishing effort (days; where 1 day = 24 h of fishing) for AIS-monitored longliners (all fleets, *n* = 3,632 grid cells) within each 1 × 1° grid cell between 2012 and 2020 was obtained from Global Fishing Watch (GFW) (globalfishingwatch.org) and mapped according to the methodology given in Queiroz et al., (2019) (Fig. S1).

### Pregnant females and embryo sampling

Pregnant females captured by the longline vessel that were already dead at gear haul back phase, were examined during fishing trips in the South Pacific Ocean in 2006 and 2007. Further observations of pregnant sharks were opportunistically made by the ship’s crew in 2011, 2012 and 2020, including the embryonic development stage (Fig. 1; Table 2). For each pregnant female, capture position, date, individual fork length (FL, ± 5 cm) and number of embryos were recorded.

**Table 2:** Summary table of dead pregnant females and embryos of shortfin mako, *Isurus oxyrinchus*, recorded during the study. FL = Fork length, SD = standard deviation.

Female FL (cm)	Litter (*n*)	Stage 0–4	Mean FL (cm)	Weight (g), (mean *±* SD)	Location	Capture date
350	15	3	53.5 ± 3.2	1697.5 ± 340.1	26º 44´ S 159º 45´ W	02/10/2006
300	11	3	48.2 ± 2.0	1763.1 ± 293.9	26º 42´ S 155º 31´ W	08/10/2006
240	10	2	32.9 ± 2.4	1611.6 ± 194.5	26º 48´ S 155º 02´ W	30/08/2006
295	14	3	49.9 ± 4.8	1760.3 ± 547.6	26º 30´ S 159º 30´ W	28/09/2006
315	11	4	-	-	29º 02´ S 99º 31´ W	06/10/2007
295	8	4	58.8 ± 1.5	2125.5 ± 424.6	30º 31´ S 103º 58´ W	11/10/2007
295	16	4	-	-	31º 15´ S 110º 51´ W	26/10/2007
310	12	4	65.3 ± 2.0	3187.7 ± 128.1	30º 51´ S 113º 37´ W	02/11/2007
295	13	4	-	-	19º 38´ S 087º 05´ W	12/11/2011
335	16	4	-	-	17º 37´ S 091º 37´ W	19/05/2012
310	10	3	-	-	15º 48’ S 107º 26’ W	14/08/2020
295	12	3	-	-	29º 38’ S 113º 14’ W	07/09/2020
370	15	4	-	-	30º 18’ S 115º 20’ W	05/10/2020
290	10	4	-	-	30º 18’ S 115º 23’ W	12/10/2020

Data collected for embryos included fork length (FL ± 0.1 cm), total weight (TW ± 0.01 g), gutted weight (GW ± 0.01 g), and liver mass. All embryos that were found to be alive were released in situ; remaining embryos were frozen on board at -20 °C until subsequent processing in the lab. Embryonic development stages were assigned based on Mollet et al., (2000) and Joung and Hsu (2005) criteria (stage 2: teeth are present in both jaws and bulging yolk stomachs; stage 3: yolk stomachs decreased, no dermal denticles yet; stage 4: complete pigmentation and adult-like teeth; Table S1 and Fig. S2 in Sup. Mat.). A subsample of yolk, liver and muscle (free from skin and cartilage) were lyophilized and frozen at -80 °C to evaluate the change in energy content during embryonic development. The gross calorific density of these tissues was determined with a Parr 6300 calorimeter©, standardized using pellets of benzoic acid (energy density: 26.4 kJ g^−1^) at regular intervals during the analysis. Total energy of each tissue was calculated as the energy density (kJ g^−1^) multiplied by the tissue total wet weight and the ratio dry: wet weight.

### Data analysis

To investigate the variation of shortfin mako body size (weight) along the longitudinal (and latitudinal) gradient, we modelled the average weight per set (haul) in a generalized additive mixed model (GAMM) framework, using the “mgcv” R package (Woods et al., 2009) to account for non-linear relationships. Briefly, we used 75% of the dataset, randomly selected as training, and set the remaining subset for model validation; and tested for collinearity among the variables using the Pearson Correlation Coefficient. Akaike Weights (wAIC) were used to select the best performing model between competing models. Two global models were constructed, one including ‘month’ and another including ‘season’, and AIC depicted the model with ‘month’ to be the best (AIC = 269.8, wAIC = 1). We also included random effects of “year” and “haul” in our global model however, ‘haul’ was not found to be significant and the final model was as follows:

1 $$\:\text{log}\left({meanWeight}_{i}\right)=f\left({month}_{i}\right)+s\left({lat}_{i}\right)+s\left({long}_{i}\right)+{uyear}_{i}+{\epsilon\:}_{i}$$

where *meanWeight* is the average weight of mako sharks per individual haul _i_, *lon* and *lat* are the geographical coordinate components (x and y, smoothed), and *uyear* is the random effect of the corresponding year of the catch, allowing for variation between years. The smoothing functions were fitted by a cubic penalised regression limited to five knots to avoid overfitting. We use a log-transformation of the average weight to meet normality assumptions.

We also analysed the variation in total energy content in embryo yolk, liver, and muscle over the period of embryonic development by performing a GAMM (Generalized Additive Mixed Model) as follows:

2 $$\:log\left({TEn}_{if}\right)=\alpha\:+f\left({ED}_{if}\right)+ELif+a+{\epsilon\:}_{if}$$

where *TEn* is the total energy content (kJ) in the tissue analysed (yolk, liver, muscle) of the embryo *i* (log-transformed) of a female *f*. *ED* is the embryonic development stage and EL is the length of embryo in cm. *a* is the random effect allowing for variation between embryos of the same batch of female shark.

The effect of the progenitor (maternal) total length on offspring size was assessed using a generalised linear model, GLM^85^ using a Poisson distribution:

3 $$\:{LS}_{i}=\alpha\:+\beta\:\times\:{FL}_{i}+{\epsilon\:}_{i}$$

where $\:{LS}_{i}$ is the litter size (number of embryos) of a female *i*. TL is the total length of the progenitor (cm). For this analysis, the data collected in this study together with the data of pregnant females available from other works^66,86,87^ were used (*n* = 85, Pacific Ocean). Graphical inspection of the residuals and random effects was used to critically evaluate the adequacy of the statistical models. All analyses and treatment of data were performed using R (R Development Core Team 2023) and the ‘mgcv_1.9-0 ^70^ and ‘nlme_3.1–163’^88^ packages.

## Results

### Spatial variation of individual body sizes and fishing effort distribution

**Fig. 2:**
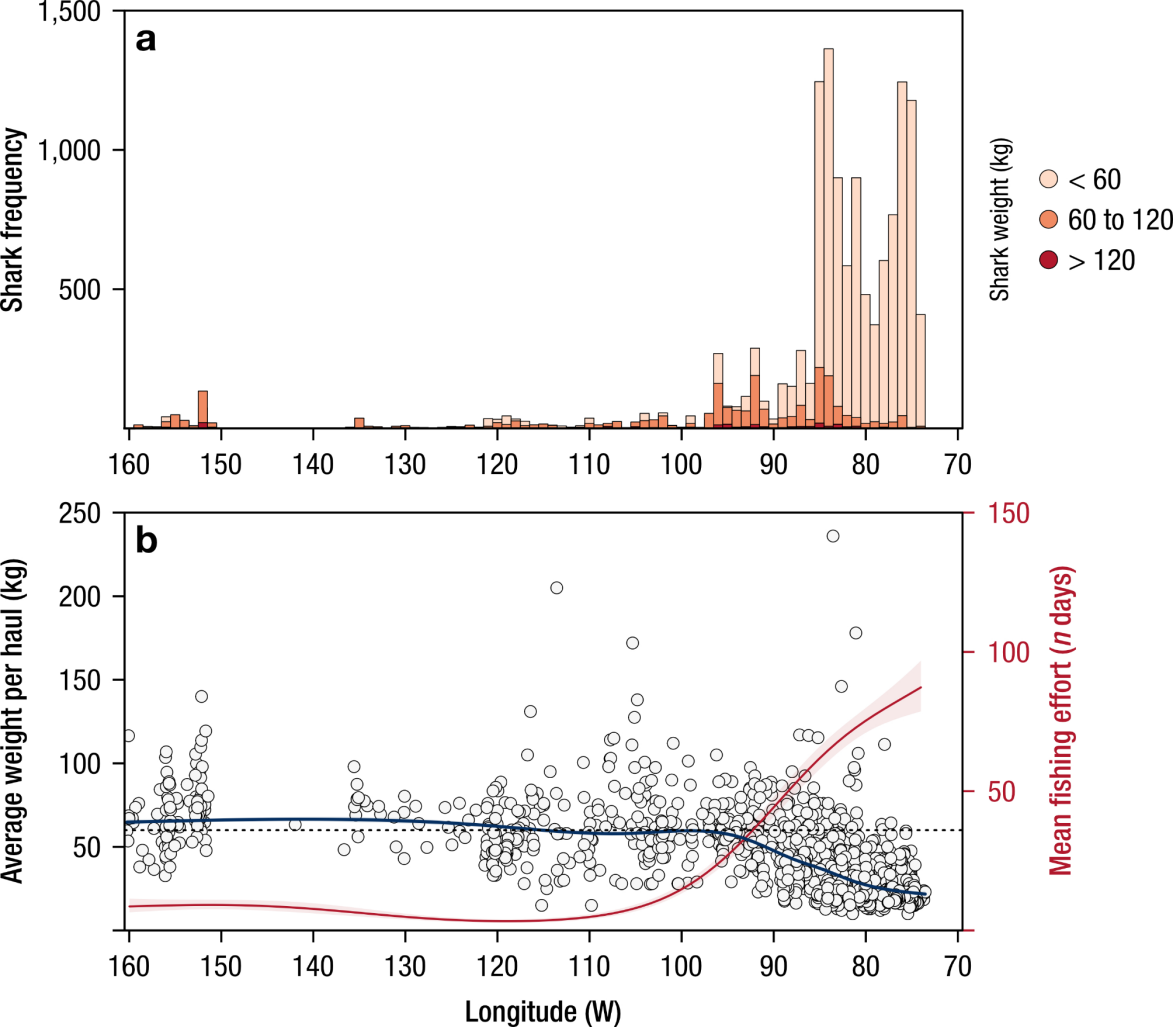
Panel (**a**) Number of individuals (immature/mature) of shortfin mako, *Isurus oxyrinchus*, found in South Pacific Ocean. Panel (**b**) GAMM (Generalized Additive Mixed Model) predictions of average weight of shortfin mako, per haul. Dark line represents the predicted mako weight varying along the range of longitudes of the study area. The red line represents mean fishing effort in days and shaded area corresponds to 95% confidence intervals. Horizontal dashed line indicates the estimated weight of size at maturity for shortfin mako (60 kg).

A total of 15,255 shortfin mako sharks were caught by the longline vessel with a mean individual weight per set of 36.9 ± 23.8 kg (mean 150 cm FL), ranging from a minimum of 7.5 kg (90 cm FL) to a maximum of 331.0 kg (315 cm FL). Although individuals of different size classes (< 60, 60–120 and > 120 kg) were observed throughout the study area (Fig. 1), juvenile sharks were found at higher frequencies in the eastern South Pacific (east of 95ºW) (Fig. 2a). Predictions from the model indicated that the average body weight was consistently larger than ~ 50 kg (~ 170 cm FL) between 160º and 100ºW but decreased significantly (*p* < 0.001) at ~ 95ºW, towards the South American continental shelf (Fig. 2b; Tables S2, S3, S4 in Sup. Mat.). Although there was a significant effect of latitude on average weight of caught sharks, a clear trend along the latitudinal gradient was not observed (Table S2 and Fig. S3 in Sup. Mat.). Finally, the best performing model explained 59.4% of the deviance (r^2^ = 0.572) and was validated by a strong correlation (Pearson) between the predicted and the actual average shortfin mako weight for the testing subset, Cor = 0.753 [CI 95% 0.7281–0.7759], t = 40.102, df = 1270, *p-value < 0.001*. The high abundance of juvenile sharks near the coast remained constant throughout each quarter of the year (Fig. S5), although smaller sharks were mainly captured during the 3rd quarter, between July and September (Fig. 2, Table S2 and Fig. S3, S5 in Sup. Mat.). Moreover, young-of-the year sharks were also occasionally observed close to the continental shelf (Fig. S6).

**Fig. 3:**
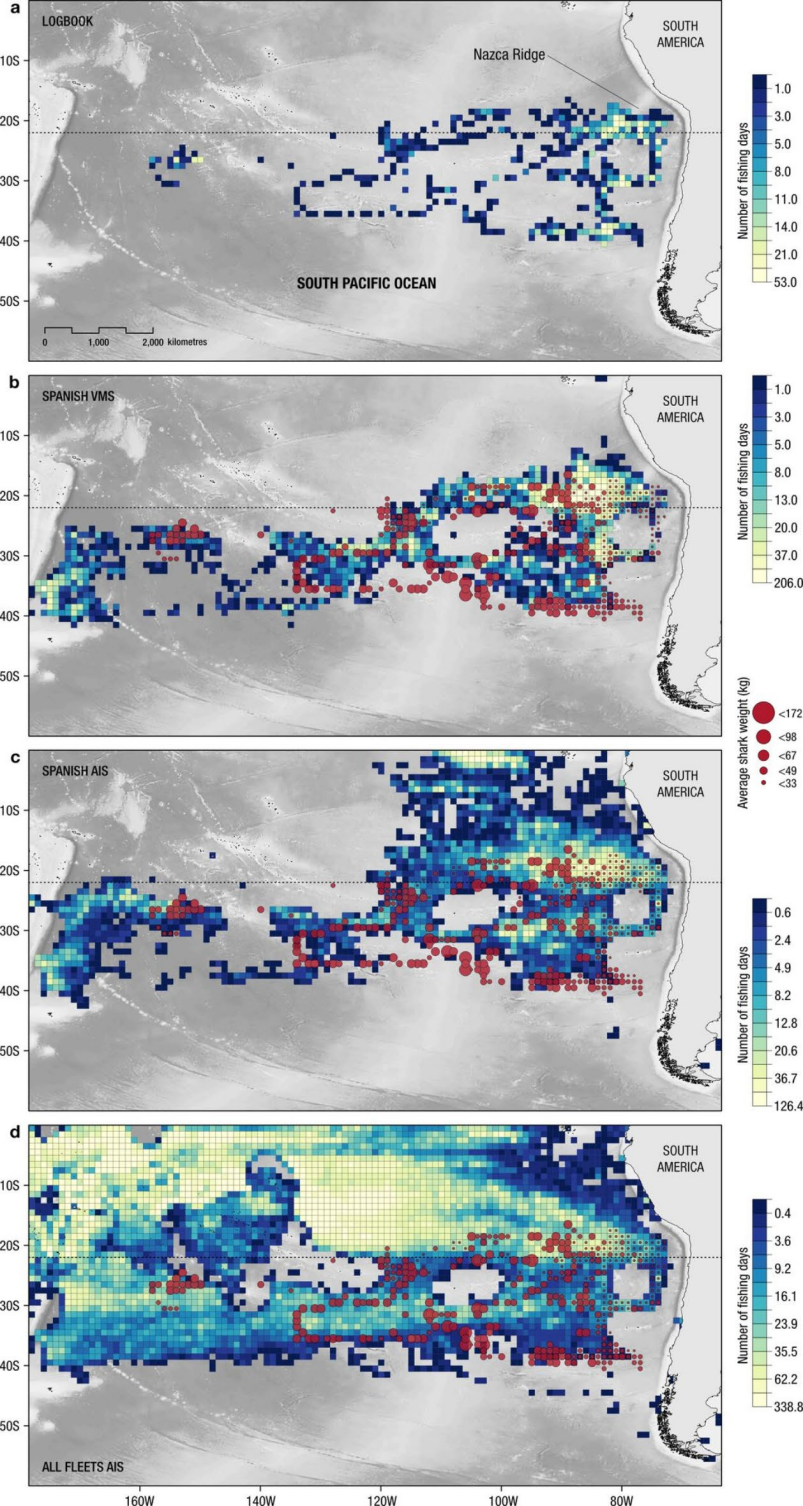
The mean fishing effort (in days; where 1 day = 24 h of fishing) distribution for logbook of Spanish vessel of this study (**a**); VMS Spanish fleet (**b**) within each 1 × 1° grid cell between 2010 and 2011 operating in the Pacific Ocean; AIS Spanish fleet (**c**) and all AIS-monitored longliners: Spain, Japan, Taiwan, Korea, and China (**d**), within each 1 × 1° grid cell between 2012 and 2020 (obtained from Global Fishing Watch, globalfishingwatch.org). Coloured circles indicates the mean size (weight) of shortfin mako, *Isurus oxyrinchus*, caught by the longline vessel between 1996 and 2009. The dark dashed line denote where the fishing effort for all nations together was generally higher north of 22ºS. Maps were created using ArcGis Pro software (version Pro 3.3).

Mean fishing effort (from the logbook catch data; 1996–2009) also showed an increase from the 100ºW meridian towards the South American coast (Fig. 2b), with higher fishing intensity observed near in the off-shelf near the Nazca Ridge (Fig. 3a). A higher fishing effort was also observed for the same general region for the Spanish longline fleet between 2010 and 2011 (VMS data; Fig. 3b) and from 2012 to 2020 (AIS data; Fig. 3c), region where the average size of shortfin makos per grid cell is smaller (Fig. 3). However, the fishing effort for all nations together (except for Spain) was generally higher north of 22ºS without a clear longitudinal trend (Fig. 3d). In the 1 × 1° grid cells where sharks were sampled (*n* = 363), 5,226 AIS fishing days were observed from 2012 to 2020, for all nations (mean of 16.0 days per grid cell); of these, Spanish longliners accounted for 3,128 days (mean of 11.0), 60% of the total. In the grid cells where the average size of the sharks was < 30 kg (*n* = 90), 1,590 and 1,208 AIS fishing days were observed for all nations and Spanish vessels, respectively. This accounted for 76% of the total fishing effort in grid cells where juvenile sharks were observed (Fig. 3).

### Embryonic development and maternal effects

**Fig. 4:**
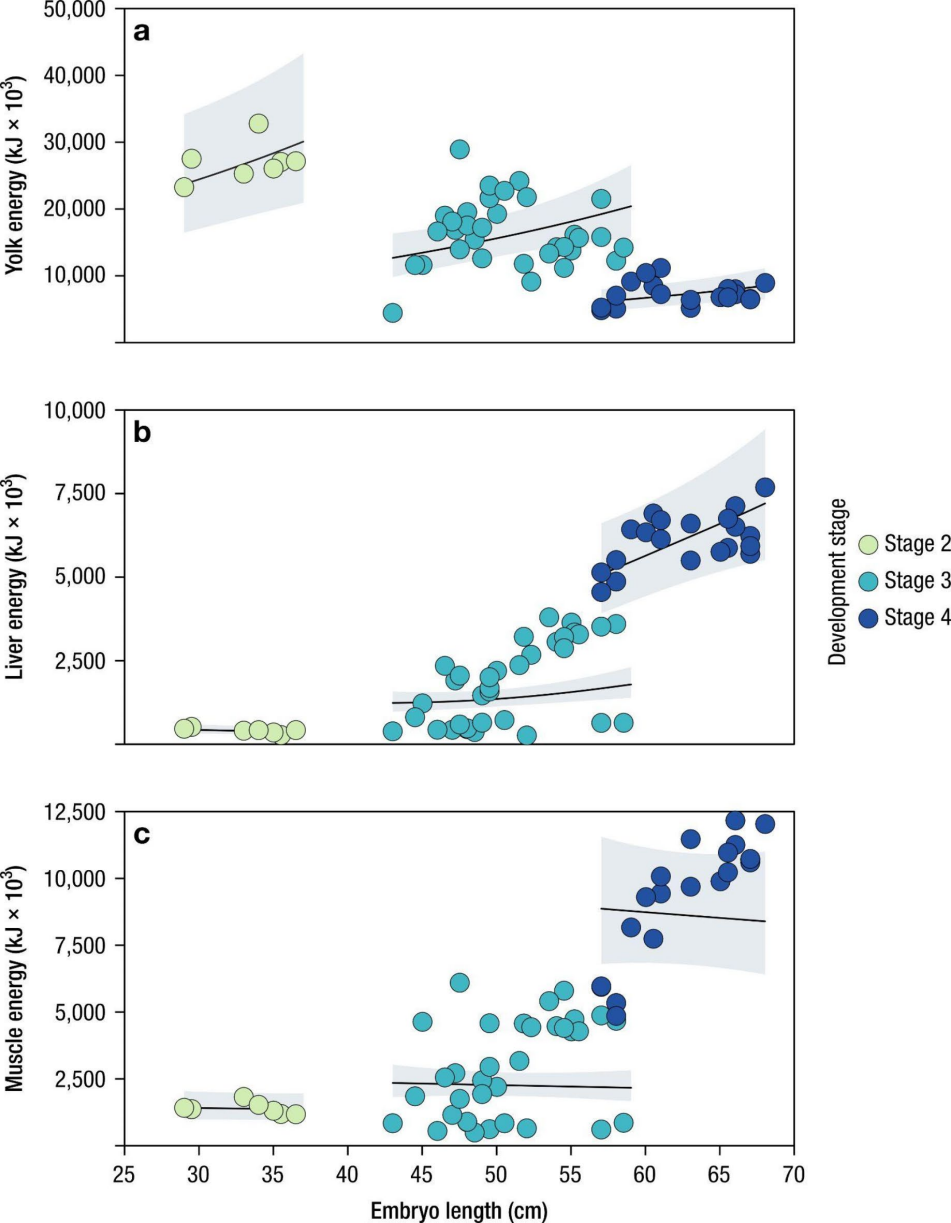
Variation of total energy (kJ) in yolk, liver and muscle along the ontogeny of shortfin mako embryos, *Isurus oxyrinchus*. Lines represent the fitted GAMM (Generalized Additive Mixed Model) for the relationship of energy content with the embryo length (Table S5). The shaded area corresponds to the 95% confidence intervals. Coloured circles correspond to embryonic development (stage 2–4).

**Fig. 5:**
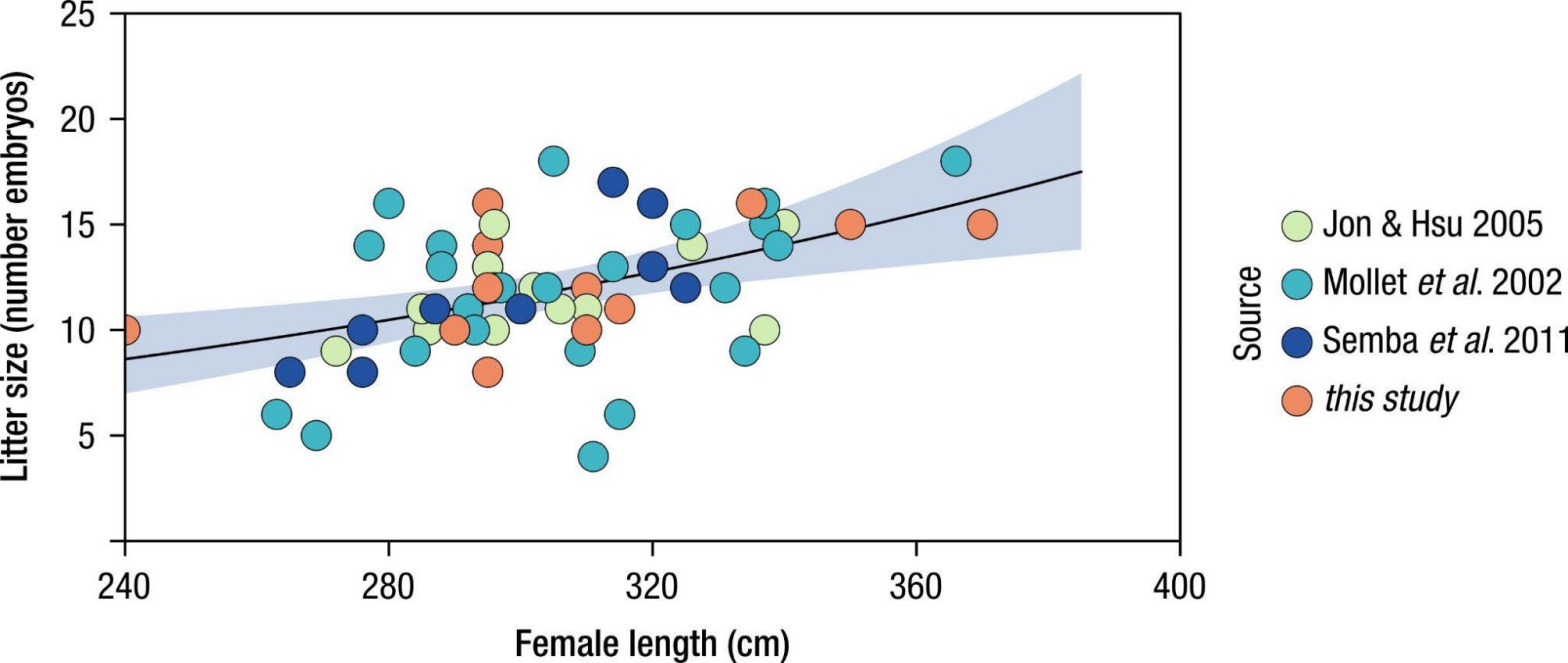
Relationship of numbers of embryos with female size of shortfin mako, *Isurus oxyrinchus*. Colours indicate source of data. Black line represents the fitted GLM (Generalized Linear Model) of the relationship of the number of offspring (litter size) with the length of the pregnant female (Table S4). The shaded area corresponds to the 95% confidence intervals.

A total of eight pregnant females were sampled from longliner catches that were brought on board as part of normal commercial operations, of 14 pregnant females recorded ranging from 150.3 kg (240 cm FL) to 538.1 kg (370 cm FL). A total of 97 embryos were observed and their development stage assessed; of these, 27 were alive and immediately released. Hence, 70 embryos were sampled, with litter size varying between eight and 16 embryos. Embryos ranged in size from 29.0 cm to 68.0 cm FL (mean = 52.4 cm) and weighed between 0.55 kg and 3.34 kg (mean = 2.03 kg) (Table 2). Embryonic stages varied between 2 and 4 (range 0–4 stages), with the mean size of the embryos increasing from 32.9 cm/1.61 kg (stage 2) to 50.8 cm/1.74 kg (stage 3) and 62.7 cm/2.76 kg (stage 4). The six pregnant females opportunistically sampled by the ship’s crew in 2011, 2012 and 2020 carried 76 embryos in total, 22 in stage 3 and 54 in stage 4. Most pregnant females (*n* = 11) were recorded between September and November, with half of the observations (*n* = 7) occurring in October. Importantly, all embryos in stage 4 were found in the central and eastern area of the study region (Fig. 1; Table 2). Yolk energy was found to significantly decrease with increased development stage (Fig. 4a) whereas the opposite was seen for the energy content in both the liver (Fig. 4b) and muscle (Fig. 4c; Table 3 and Table S5 in Sup. Mat.), despite not being significant. Finally, larger females produced more offspring (Fig. 5, Table S6).

**Table 3:** Morphometric and bioenergetic data of shortfin mako embryos, *Isurus oxyrinchus*, by stage of development. Mean values given with standard deviation. ED = energy density; FL = Fork Lenght.

Stage	Sex	*n*	FL (cm)	Weight (g)	Yolk (g)	Liver (g)	ED yolk (Kj/g)	ED liver (Kj/g)	ED muscle (Kj/g)
2	Female	5	33.7 ± 1.5	1556 ± 149.3	1395.8 ± 172	10.8 ± 2.2	30515.88 ± 457.7	29292.73 ± 856.46	16664.87 ± 485.34
	Male	5	32 ± 3.2	1667.2 ± 254.1	1523.4 ± 247.2	15.4 ± 1.6	30787.34 ± 543.5	29551.42 ± 1149.76	16765.29 ± 374.47
3	Female	19	51.3 ± 4.7	1754.5 ± 463.8	950.6 ± 253.2	68.9 ± 45.6	30399.86 ± 543.5	35392.04 ± 871.95	20230.9 ± 643.92
	Male	21	50.3 ± 4	1717.6 ± 389.4	914.7 ± 267.2	79.4 ± 43.8	30358.69 ± 471.54	35217.56 ± 1036.38	20181.11 ± 869.02
4	Female	10	61.8 ± 3.9	2607.5 ± 667.5	414.7 ± 164	223.5 ± 44.1	30385.88 ± 569.86	36797.44 ± 346.02	21960.56 ± 986.17
	Male	10	61.1 ± 3.7	2543.8 ± 563	309.6 ± 169.2	193.5 ± 32.7	30030.24 ± 936.38	36716.27 ± 517.56	21,653 ± 828.43

## Discussion

In this study, we provide evidence of a longitudinal gradient in size distribution and embryo development, which was persistent through sampling years, indicating a potential nursery area for shortfin mako in the open-ocean waters of the South-east Pacific. We have also quantified for the first time, the net energetic transfer from the mother to the embryo for shortfin mako. Of particular concern is that our results also indicate that a hotspot for longline fisheries likely spatially overlapped the potential nursery. Taken together, our findings provide fundamental knowledge on the spatial and reproduction biology of shortfin makos, revealing an increased fishing risk for key life-stages in the South-east Pacific.

The average body size of shortfin makos caught by the commercial vessel showed a clear longitudinal gradient, decreasing from west to east, particularly from the 95ºW meridian towards the South American coast. In agreement with the observed longitudinal size gradient, the embryonic development of pregnant females also followed a longitudinal gradient with less developed embryos observed in the south-west, when compared to more developed embryos in the south-eastern Pacific Ocean. These observations indicate spatial segregation of different components of the shortfin mako population, suggesting a potential long-distance female migration from open-ocean areas in the west to near shelf areas in the east, albeit individual movements were not observed in this study. Observations of pregnant shortfin mako in late stages of embryonic development towards the south-east Pacific could be linked to females searching for more productive waters in which to give birth, providing an abundance of potential prey species with body sizes appropriate to neonates^13,25,89^. The migration of mature or pregnant females over deep-ocean to coastal areas to give birth has been previously suggested in large sharks. For example, pregnant females of sand tiger shark, *Carcharias taurus*, migrated southwards to their parturition sites and aggregated in southern Queensland, in Australia^90^; sexually mature females of white shark, *Carcharodon carcharias*, remained in offshore waters during their 2-year migration cycle, before travelling to the pupping area along the Mexican coast^91^. During this offshore period, pregnant females may be less vulnerable to fishing since mature specimens are caught less frequently. For example, out of 19,905 shortfin mako females caught between 1993 and 2019 across the Atlantic, Indian, and Pacific Oceans by the Spanish surface longline fleet, only 92 (0.4%) were pregnant^92^.

The present work suggests, for the first time, the existence of a potential nursery for shortfin mako sharks in the South Pacific. In the North Pacific, at least two coastal nurseries, separated by ~ 500 km of coastline, were described in the California coast^35,70–72^ in the Southern California Bight, USA, and Bahia Sebastian Vizcaino, Mexico, with previous studies suggesting that juveniles remain in these regions, primarily foraging, before moving into the open ocean habitat as they mature^59,93^. Likewise, possible nursery areas for other oceanic sharks have also been identified, such as blue shark *Prionace glauca* in the south-eastern^7^ and white shark *Carcharodon carcharias* in the north-eastern Pacific^94^, respectively, where juvenile sharks occupy coastal areas until they become sub-adults. The potential nursery identified in the present study is presumably linked to the increased foraging opportunities provided by the Humboldt Current^95^. The high productivity in this region is caused by the strong seasonal upwelling that enables the development of important pelagic fishes and cephalopods^96^, which are the preferred prey of juvenile shortfin makos in the area^97^.

We observed substantial overlap between the distribution of shortfin makos and longline fishing effort, especially in the area where juvenile makos were recorded. Whilst the overlap with all fleets combined was moderate to high, when the Spanish fleet was mapped separately, we observed the highest overlap, suggesting that the fishery has historically concentrated its activity in areas where there is a higher presence of juveniles/immatures. Our results indicate that areas with smaller shortfin makos are expected to be exposed to more intense fishing activity, which could affect the population if management measures are not put in place to mitigate this high level of spatial overlap. Major high-seas fishing activities are centred on ecologically important shark hotspots worldwide^2^. For example, in North Atlantic blue sharks and shortfin mako have on average 76% and 62% of their monthly space use, respectively, overlapped by longline fishing effort^1,2^, with greater fishing-induced mortality of pelagic sharks where spatial overlap intensity was greater^49^. Importantly, even though logbook data and VMS/AIS locations did not overlap temporally, fishing hotspots tend to remain constant over the years^2,49,50,98^. For example, the fishing hotspot for the Spanish fleet in the eastern South Pacific remained relatively stable, between 2010 and 2019 (Fig. 3a, b,c).

Most shark species segregate by size and/or sex at some point of their life cycle and the degree of size and sex segregation shapes their population structure and informs the appropriate scale for population management^99,100^. Critically, the determination of ecological aspects, such as potential aggregation ‘hotspots’, essential habitats, and spatiotemporal interactions with fisheries for different life stages, are essential for fishery management^101–103^. Recent tagging and recovery studies estimated that fishing activity was responsible for more than half of total mortality of juvenile shortfin makos^82^, where only 20% of the male population and 2.1% of the female population would reach the maturation in North Atlantic^82^. Other studies based on fisheries independent data resulted in 30% of tagged makos harvested^2,74^.

While it has been shown for other shark species (mainly *Carcharhinus* spp.)^25,38^, the present work provides the first information on the energetic strategy of shortfin mako embryos. The mother provides energy in the form of yolk through unfertilized eggs that embryos store in large quantities in their stomachs and metabolize progressively, increasing the energy content in the livers. In addition, larger mothers produced greater numbers of embryos than smaller. The variation of energy content in the main tissues (yolk, liver and muscle) of embryos suggests a significant net positive transfer of energy from yolk to liver and muscle but with yolk energy remaining in embryos near term (stage 4). Therefore, the energy transfer provided by the mother is a resource available at parturition, with the pups directly utilizing these reserves in conjunction with independent foraging^14,22^. Whereas in large adult sharks, the liver acts as a storage for lipid reserves, which are used in reproduction, migration and during periods of prey limitation^14,22,104^, in neonate sharks, several authors have proposed that the enlarged liver might act as an energy reserve to be utilized after birth^14,67,68,105,106^. The near-term pups of *C. taurus*, longfin mako *Isurus paucus*, and porbeagle shark *Lamna nasus* have larger livers than small prenatal and neonatal sharks^22,67,105,106^. In the early stages of neonatal growth, where individuals have little hunting experience and are losing yolk content in the stomach, it is possible that they become more vulnerable to fishing, being attracted to baits used by fishermen, mainly mackerel and squid^107^.

While neonates and young juveniles are important for population recovery, the conservation of older life stages may be equally or more critical^39,82,108^. Thus, effective shark management should integrate nursery protection with strategies targeting older individuals across broader habitats^9,39,100^. Previous work suggests that time-area closures of fishing for nursery populations of highly mobile shark species may be an important tool of management, but not without additional measures such as catch limits^26,39,101^. However, Regional Fisheries Management Organizations (RFMOs), particularly ICCAT, have been criticized for their permissive regulation and low conservation priority regarding oceanic sharks^109^, which is exemplified by a lack of effective management in reducing bycatch mortality of protected and/or endangered sharks^53^.

The South Pacific Ocean is a region heavily fished for large pelagic species including oceanic sharks^2^. South Pacific makos have been caught by longline fisheries since the 1950s, but catch records only started to be reported in the 1990s^79^. In fact, the first attempt at undertaking an assessment of the southwest Pacific stock in 2022 concluded that it was not robust enough for providing management advice^79^. The assessment of the South Pacific shortfin mako stock highlights the poor representation of mature females in commercial fishing data, which could support the suggestion that during pregnancy they are less vulnerable (see above; Large et al., 2022), or that data were not fully reported^48^. It is widely acknowledged that strict prohibitions and precautionary science-based catch limits are needed urgently for oceanic sharks to avert population collapses, avoid the disruption of ecological functions and promote species recovery^48,51,52,54^. Accurate assessments of populations and conservation status of shortfin mako remain difficult due to its trans-migratory movements, crossing international boundaries^55,109^, and although it is caught in some regions in relatively high numbers compared to other oceanic sharks^53^, it is poorly monitored and faces unregulated fisheries worldwide^48,109^.

This study identifies a critical potential nursery area for shortfin mako sharks which overlaps with a global fishing hotspot. This overlap raises significant concerns about the potential overexploitation of key life stages within the population, such as pregnant females and juveniles. Nonetheless, future efforts should incorporate fisheries-independent data (e.g., tracking movements and habitat use of juvenile makos), as well as additional size- and species-specific catch data from broader range of fishing vessels than was analysed in this study to more precisely delineate the potential nursery area. Accurate data of different types can then be used to consider the extent of spatial protection needed for this potential nursery. A high seas marine protected area (MPA) may be a useful conservation tool to protect young shortfin makos during their early life history or/and pregnant females in this exploited region of the south Pacific^110^.

Importantly, the present study was limited not only by the low number of pregnant females captured to fully assess the reproductive strategy of shortfin makos in the South Pacific Ocean, but also by the fact that only the logbook data from a single longliner was analysed. On the other hand, we are defining a potential open ocean nursery for this species solely based on fishery dependent data. Nonetheless, despite these shortcomings this study contributes with critical findings and invaluable data to further investigate the presence of putative nursery ground for shortfin mako.

In conclusion, we have shown a high concentration of juvenile sharks in deep, open-ocean waters off the shelf of South America (i.e., off Peru and Chile), and we described a net transfer of energy from mothers to embryos, observed throughout the embryonic development stages. Furthermore, pregnant females with more developed embryos were generally observed further east. It is of critical concern that this area also appears to overlap spatially with some of the highest levels of fishing activity in the region, which has implications for the management of the fishery. For example, since the overlap is likely driven primarily by vessels from a single country (Spain), implementing urgent management measures – such as establishing catch limits or introducing spatial protection zones – to mitigate fishing-induced mortality of juveniles of mako sharks in the South Pacific could be relatively straightforward.

## Electronic supplementary material

Below is the link to the electronic supplementary material.

Supplementary Material 1

## Data Availability

The datasets used and/or analysed during the current study are available from the corresponding author on reasonable request. Part of data generated or analysed during this study are included in this published article [and its supplementary information files]. The fishing effort data was extracted from Global Fishing Watch portal (https://globalfishingwatch.org/).
