# Supplementary material for: Discovery of a potential open ocean nursery for the endangered shortfin mako shark in a global fishing hotspot
Source: Sci Rep. 2025 Jan 16;15:2190. doi: 10.1038/s41598-025-85572-4 (PMC11739380; doi:10.1038/s41598-025-85572-4)
Supplement: Supplementary file 1 — Supplementary Material 1 [file 41598_2025_85572_MOESM1_ESM.docx]

**Discovery of a potential open ocean nursery for the endangered shortfin mako shark in a global fishing hotspot**

Gonzalo Mucientes^a,b,c*^, Alexandre Alonso-Fernández^c^, Marisa Vedor^a,b^, David W. Sims^d,e^, Nuno Queiroz^a,b,*^

^a^Centro de Investigação em Biodiversidade e Recursos Genéticos, CIBIO-InBIO, Universidade do Porto. Campus Agrário de Vairão, r/ Padre Armando Quintas, 4485-661 Vairão, Portugal.

^b^BIOPOLIS Program in Genomics, Biodiversity and Land Planning, CIBIO, Campus de Vairão, 4485-661 Vairão, Portugal

^c^Instituto de Investigaciones Marinas (IIM), CSIC, Eduardo Cabello 6, 36208 Vigo, Spain

^d^Marine Biological Association, The Laboratory, Citadel Hill, Plymouth PL1 2PB, UK.

^e^Ocean and Earth Science, University of Southampton, National Oceanography Centre Southampton, Waterfront Campus, Southampton SO14 3ZH, UK

* Corresponding authors: gmucientes@iim.csic.es/nuno.queiroz@cibio.up.pt

**Table S1.** Embryonic development of shortfin mako, *Isurus oxyrinchus*, stages criteria.

| Embryonic stage | Description |
| --- | --- |
| 0 | Just after mating, fertilised |
| 1 | Embryos are visible, external gill filaments, no teeth. |
| 2 | Teeth are present in both jaws and bulging yolk stomach of the embryos reached their maximum size. |
| 3 | The bulging yolk stomachs decreased, no dermal denticles yet. |
| 4 | Complete pigmentation and reabsorbed gills, new adult-like teeth (presence of free teeth in the stomach content) and formed caudal fin. |

**Table S2**. Best generalised additive mixed model (GAMM) results of mako shark average weight, including the parametric coefficients: *Std. Error* = standard error; *Std. Dev* = standard deviation; *t value:* The test statistic and *Pr(>|t|)*: The p-value associated with the test; and the approximate significance of smooth terms: edf = estimated degrees of freedom; F = F statistic for significance of smooth term; p-value: The p-value associated with the test.

|  | Estimate | Std. Error | t value | Pr(>\|t\|) |
| --- | --- | --- | --- | --- |
| factor(month)1 | 3.88 | 0.079 | 48.92 | <0.0001 |
| factor(month)2 | 3.87 | 0.064 | 60.62 | <0.0001 |
| factor(month)3 | 3.93 | 0.055 | 71.65 | <0.0001 |
| factor(month)4 | 3.86 | 0.061 | 62.89 | <0.0001 |
| factor(month)5 | 3.69 | 0.058 | 63.4 | <0.0001 |
| factor(month)6 | 3.42 | 0.055 | 61.92 | <0.0001 |
| factor(month)7 | 3.44 | 0.055 | 62.24 | <0.0001 |
| factor(month)8 | 3.43 | 0.053 | 64.35 | <0.0001 |
| factor(month)9 | 3.35 | 0.065 | 51.76 | <0.0001 |
| factor(month)10 | 3.65 | 0.069 | 53.06 | <0.0001 |
| factor(month)11 | 3.55 | 0.088 | 40.51 | <0.0001 |
| factor(month)12 | 3.60 | 0.094 | 38.42 | <0.0001 |
|  |  |  |  |  |
|  | edf | Ref.df | F | p-value |
| s(long) | 3.360 | 3.774 | 35.94 | <0.0001 |
| s(lat) | 2.913 | 3.443 | 7.235 | 0.0004 |
| s(year,bs="re") | 1.30E-06 | 1 | 0 | 0.0294 |

**Table S3.** GAMM model selection between both temporal fixed effects (‘month’ and ‘season’). *logLik* is the log likelihood of the model;*AICc* is Akaike Information Criterion corrected for small sample sizes; *delta* is the difference between the AIC value of a given model and the AIC value of the best model (the one with the lowest AIC) and *weight* is the AIC weight, the probability that a model is the best among the set.

|  | logLik | AICc | delta | weight |
| --- | --- | --- | --- | --- |
| log(weight) ~ factor(month)-1 + s(long1, k = 5)+ s(lat1, k = 5)+s(haul,bs="re")+s(year,bs="re") | -113.315 | 269.8 | 0 | 1 |
| log(weight) ~ factor(season)-1 + s(long1, k = 5)+ s(lat1, k = 5)+s(haul,bs="re")+s(year,bs="re") | -133.379 | 290.7 | 20.86 | 0 |

**Table S4**. GAMM best performing model selection after excluding non-significant variable ‘*haul’. logLik* is the log likelihood of the model; *AICc* is Akaike Information Criterion corrected for small sample sizes; *delta* is the difference between the AIC value of the model and the AIC value of the best model (the one with the lowest AIC) and *weight* is the AIC weight, the probability that a model is the best among the set.

|  | logLik | AICc | delta | weight |
| --- | --- | --- | --- | --- |
| log(weight) ~ factor(month)-1 + s(long1, k = 5)+ s(lat1, k = 5)+s(year,bs="re") | -113.559 | 269.7 | 0 | 0.521 |
| log(weight) ~ factor(month)-1 + s(long1, k = 5)+ s(lat1, k = 5)+s(haul,bs="re")+s(year,bs="re") | -113.315 | 269.8 | 0.17 | 0.479 |

**Table S5**. Summary table of the GAMM (Generalized Additive Mixed Model) fitted to the relationship between energy content in yolk, liver and muscle with the length of embryos of shortfin mako, *Isurus oxyrinchus*. Std. Error = standard error; Std. Dev = standard deviation; edf = estimated degrees of freedom; Weight = predicted weight of shortfin mako.

| **YOLK** |  |  |  |  |
| --- | --- | --- | --- | --- |
| **Coefficients** | **Estimate** | **Std. Error** | **t** | ***p*-value** |
| Intercept | 10.7779 | 0.2828 | 38.1065 | <0.0001 |
| Development Stage 3 | -1.0420 | 0.2841 | -3.6679 | 0.0351 |
| Development Stage 4 | -2.1846 | 0.3905 | -5.5949 | 0.0113 |
| Length | -0.2866 | 0.1072 | -2.6744 | 0.0099 |
| **Random effects** | Female | Residual |  |  |
| Std. Dev | 0.1477 | 0.2539 |  |  |
| **LIVER** |  |  |  |  |
| **Coefficients** | **Estimate** | **Std. Error** | **t** | ***p*-value** |
| Intercept | 5.9851 | 0.6438 | 9.2962 | <0.0001 |
| Development Stage 3 | 1.3454 | 0.7330 | 1.8356 | 0.1638 |
| Development Stage 4 | 2.4824 | 0.7960 | 3.1186 | 0.0525 |
| Length | -0.0037 | 0.1889 | -0.0196 | 0.9845 |
| **Random effects** | Female | Residual |  |  |
| Std. Dev | 0.5600 | 0.2210 |  |  |
| **MUSCLE** |  |  |  |  |
| **Coefficients** | **Estimate** | **Std. Error** | **t** | ***p*-value** |
| Intercept | 7.1455 | 0.6123 | 11.6697 | <0.0001 |
| Development Stage 3 | 0.5748 | 0.6649 | 0.8644 | 0.4509 |
| Development Stage 4 | 1.9725 | 0.7958 | 2.4787 | 0.0894 |
| Length | 0.0480 | 0.1600 | 0.3001 | 0.7653 |
| **Random effects** | Female | Residual |  |  |
| Std. Dev | 0.4847 | 0.3547 |  |  |

**Table S6**. Summary table of the GLM (Generalized linear model) fitted to the relationship between litter size (number of embryos) and body size (total length, cm) of pregnant female shortfin makos, *I. oxyrinchus.* Std. Error = standard error.

| **Parametric coefficients** | **Estimate** | **Std. Error** | **Z** | ***p*-value** |
| --- | --- | --- | --- | --- |
| Intercept | 0.9818 | 0.4569 | 2.149 | 0.0316 |
| TL | 0.0049 | 0.0015 | 3.299 | 0.0009 |

**Figure S1**. Mean fishing effort (in days; where 1 day = 24 h of fishing) distribution AIS-monitored longliners from China (a), Taiwan (b), South Korea (c) and Japan (d), within each 1 × 1° grid cell between 2012 and 2019 (obtained from Global Fishing Watch, globalfishingwatch.org), and mean size (weight, circles) of shortfin mako, *Isurus oxyrinchus*, caught by surface longline vessels between 1996 and 2009.


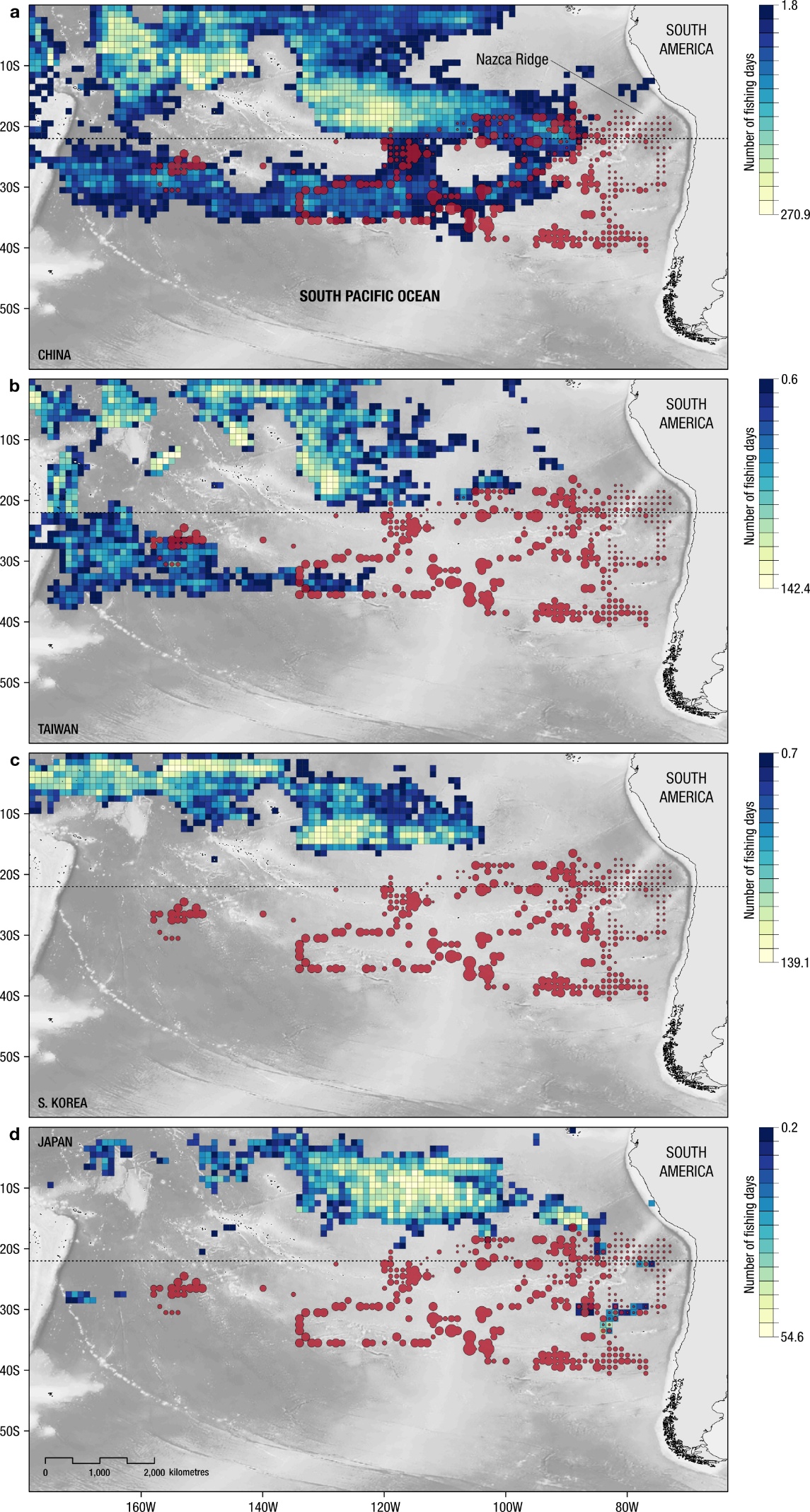


**Figure S2**. Different stages of development of embryos belonging to shortfin mako, *Isurus oxyrinchus* found in this study during fishing trips; (a) stage 2, (b) stage 3 and (c) stage 4. Newborn free-swimming shortfin mako of 68 cm total length captured and returned to the sea by the longliner vessel (d).


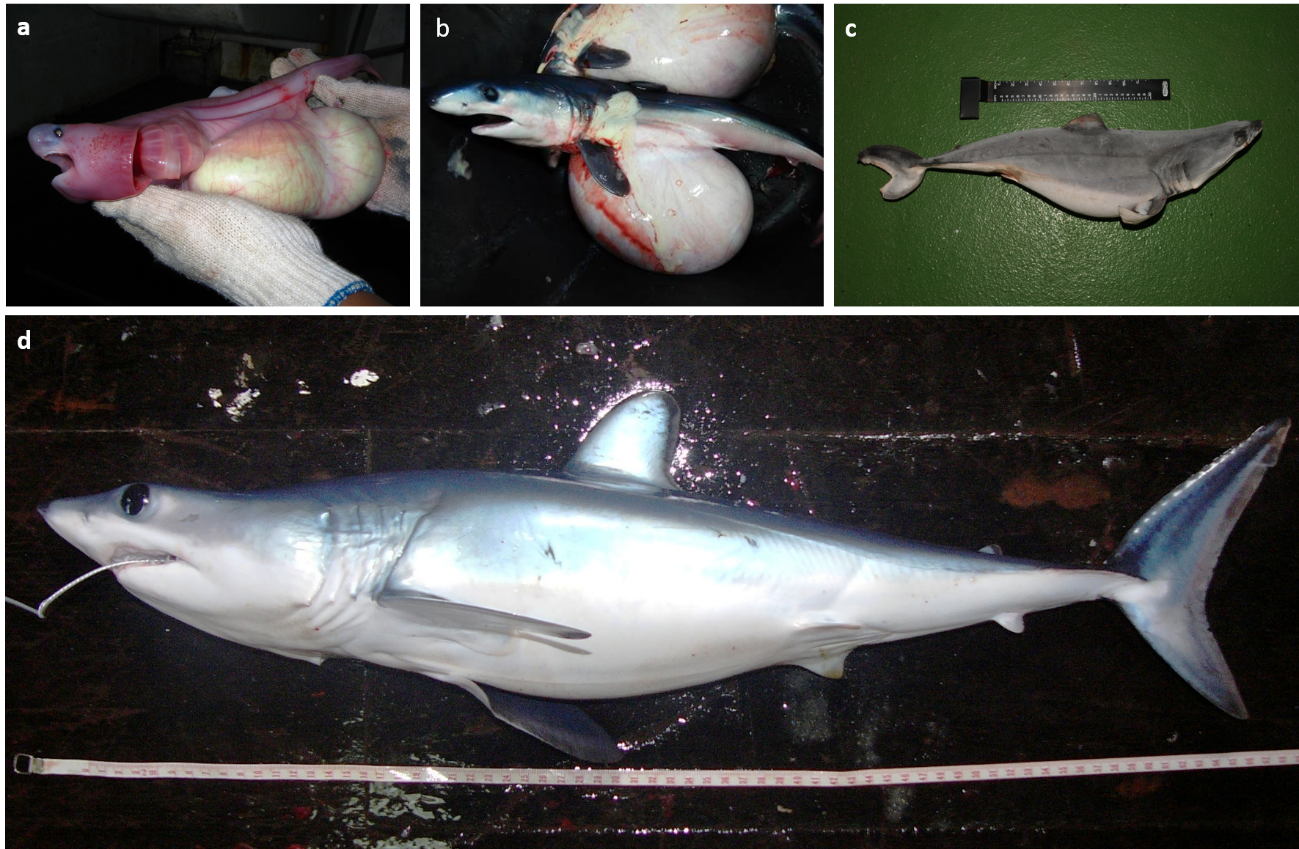


**Figure S3**. Response curves from the best performing model of average mako weight per haul, with the response on the y axis (smoothed) and predictor variable range on the x axis. Shaded areas represent the 95% confidence intervals.


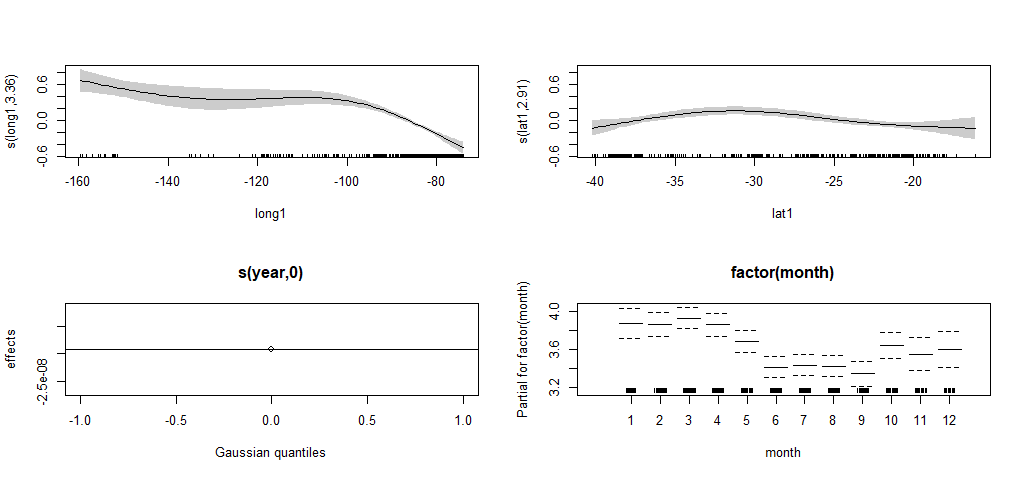


**Figure S4.** Histogram of size frequency distribution of shortfin mako (*Isurus oxyrinchus*) captured along the longitudinal gradient. At longitudes smaller than 80 degrees juvenile individuals are predominant and mature individuals are absent. The black line indicates the average weight for mature individuals for both sexes (177 cm, ~60kg).


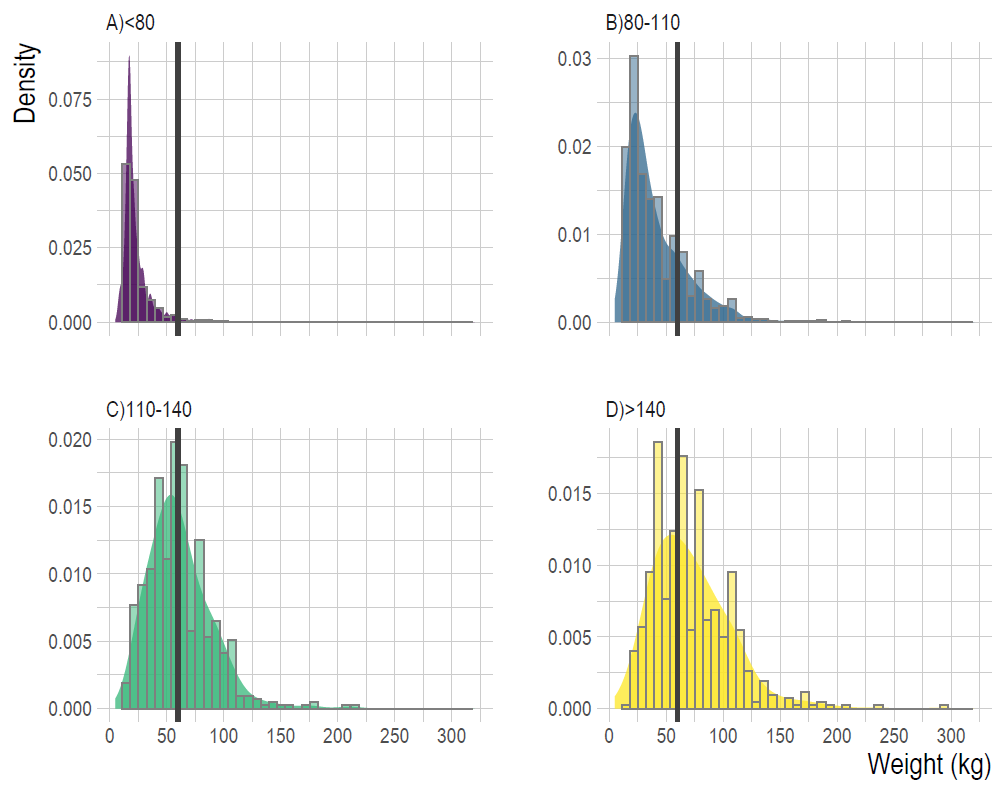


**Figure S5.** Box plot showing the longitudinal trend in the average weight of shortfin makos (*Isurus oxyrinchus*) caught in each quarter of the year (smaller individuals are closer to the South American continental shelf).

**
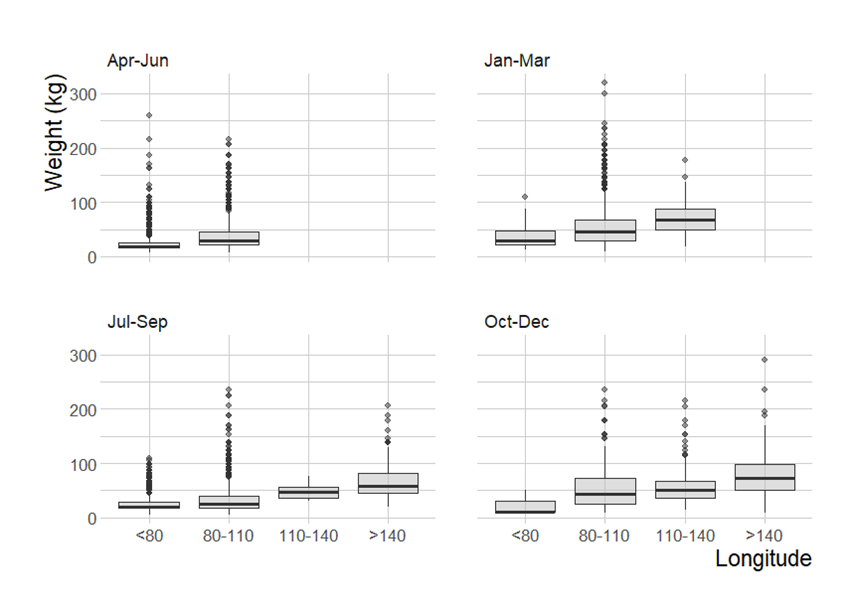
**

**Figure S6.** CPUE (catch per unit effort) variation of small juveniles captures of shortfin mako, *Isurus oxyrinchus*, along the longitude (the majority individuals are closer to the South American continental shelf).

**
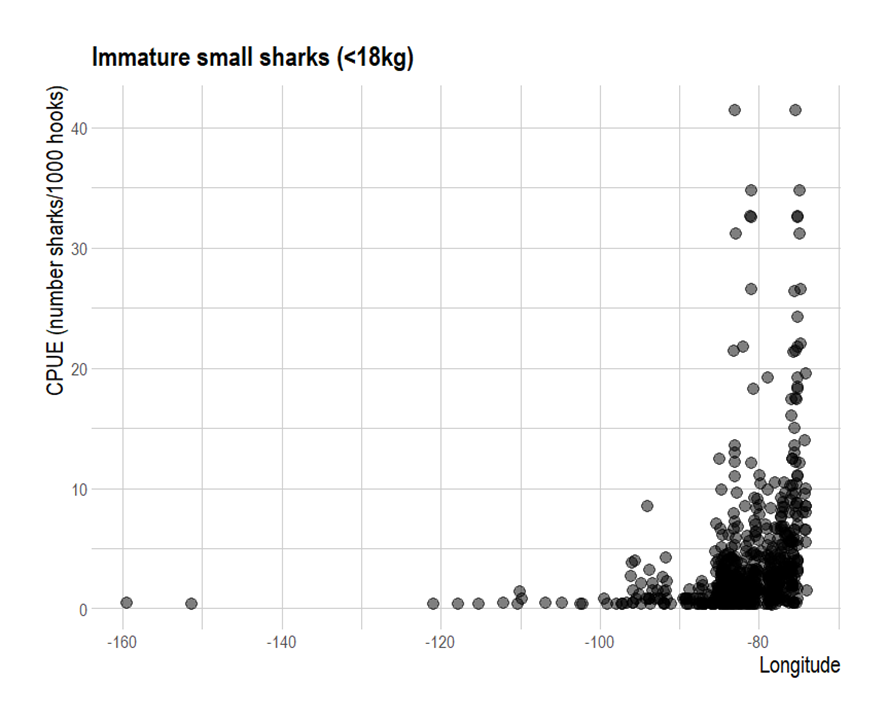
**
